# Flexible, Suturable, and Leak-free Scaffolds for Vascular Tissue Engineering Using Melt Spinning

**DOI:** 10.1021/acsbiomaterials.3c00535

**Published:** 2023-07-25

**Authors:** Julia Fernández-Pérez, Kenny A. van Kampen, Carlos Mota, Matthew Baker, Lorenzo Moroni

**Affiliations:** Department of Complex Tissue Regeneration, MERLN Institute for Technology-Inspired Regenerative Medicine, Maastricht University, Universiteitssingel 40, 6229ER Maastricht, The Netherlands

**Keywords:** vascular grafts, scaffolds, fiber orientation, regenerative medicine, additive manufacturing

## Abstract

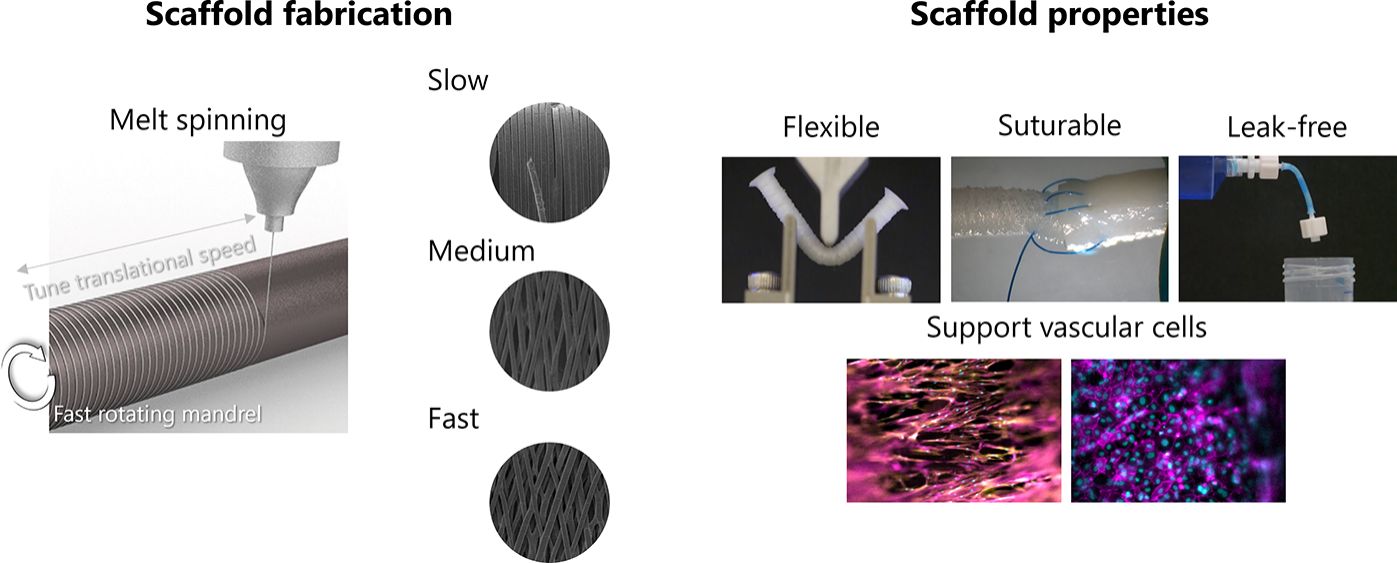

Coronary artery disease affects millions worldwide. Bypass surgery remains the gold standard; however, autologous tissue is not always available. Hence, the need for an off-the-shelf graft to treat these patients remains extremely high. Using melt spinning, we describe here the fabrication of tubular scaffolds composed of microfibers aligned in the circumferential orientation mimicking the organized extracellular matrix in the tunica media of arteries. By variation of the translational extruder speed, the angle between fibers ranged from 0 to ∼30°. Scaffolds with the highest angle showed the best performance in a three-point bending test. These constructs could be bent up to 160% strain without kinking or breakage. Furthermore, when liquid was passed through the scaffolds, no leakage was observed. Suturing of native arteries was successful. Mesenchymal stromal cells were seeded on the scaffolds and differentiated into vascular smooth muscle-like cells (vSMCs) by reduction of serum and addition of transforming growth factor beta 1 and ascorbic acid. The scaffolds with a higher angle between fibers showed increased expression of vSMC markers alpha smooth muscle actin, calponin, and smooth muscle protein 22-alpha, whereas a decrease in collagen 1 expression was observed, indicating a positive contractile phenotype. Endothelial cells were seeded on the repopulated scaffolds and formed a tightly packed monolayer on the luminal side. Our study shows a one-step fabrication for ECM-mimicking scaffolds with good handleability, leak-free property, and suturability; the excellent biocompatibility allowed the growth of a bilayered construct. Future work will explore the possibility of using these scaffolds as vascular conduits in *in vivo* settings.

## Introduction

Cardiovascular diseases are one of the leading causes of mortality worldwide and associated with around 28% of deaths in The Netherlands alone.^1^ When the coronary artery is obstructed by an atherosclerotic plaque, the blood supply to the heart is compromised. To restore blood flow, a stent can be placed or a bypass surgery performed. Stents fail over time via intima hyperplasia and need to be replaced. In bypass surgeries, a segment of the saphenous vein or the mammary artery is harvested and implanted. This procedure requires healthy tissue to be removed, which is not always available and can lead to site morbidity.^2^ Although synthetic grafts are available for large-diameter vessels, these fail when intended to use for small-caliber (<6 mm diameter) arteries, such as the coronaries.^3^ Hence, the need for an off-the-shelf graft to treat these patients remains extremely high.

Tissue-engineered vascular grafts obtained by self-assembly techniques are highly biomimetic, but require lengthy and costly culturing times. L’Heureux et al. created tubular grafts from sheets of matrix secreted by fibroblasts by either rolling^4^ or threading.^5^ Pioneering work by Niklason et al., currently in phase III clinical trials through Humacyte, is based on the dynamic culturing of cells on fast-degrading polymeric scaffolds that are decellularized upon maturation.^6,7^ Another method is to subcutaneously implant a cylindrical template with engineered surface properties able to influence the formation of an engineered fibrous capsule via actively steering the foreign body response. The templates are then retrieved to remove the formed biological construct.^8,9^ Another important strategy for the fabrication of vascular grafts is *in situ* tissue engineering, by which acellular scaffolds are implanted and the body can repopulate and remodel them. This strategy removes the necessity of prior cell culturing, terminal sterilization can be performed without problems, scaffolds can be readily available to the surgeon, and the costs are remarkably lower.

The main strategies to obtain grafts for implantation are fabricating highly porous scaffolds via solvent casting from a myriad of materials, such as silk fibroin,^10,11^ elastin-like recombinamers,^12^ or poly(glycerol sebacate) (PGS).^13^ Although easy to fabricate, these scaffolds do not recapitulate the ECM structural organization of the vessel. Electrospinning can be used to obtain fibrous scaffolds with fibers ranging from the nanoscale to the microscale. Controlling the alignment of these fibers in a small-diameter collecting mandrel is challenging.^14^ Melt spinning offers advantages to electrospinning as no solvents need to be used and to melt electrowriting as no voltage needs to be applied, so less problems with repulsion electrostatic forces occur. We have recently described a custom-built four-axis 3D printer ideal for the production of tubular scaffolds from thermoplastic polymers with control over the fiber size and fiber alignment in the circumferential orientation.^15^ Such scaffolds can mimic the orientation of the ECM in the tunica media of arteries. This layer is populated by smooth muscle cells (SMCs) and their smooth muscle fibers composed of elastin and collagen and disposed in lamellae and circularly arranged around the vessel, thus contributing to the contractile properties of arteries. In our previous work, the impact of the printing parameters on scaffold properties was not explored.

In the current study, we describe the fabrication of tubular scaffolds with different fiber alignments, which have robust mechanical properties and are leak-free and suturable. We study the effect of these properties on the cell behavior of hMSC differentiated toward SMC-like cells. Endothelial cells were also seeded to obtain a construct comprising the intima and media layers of the artery.

## Materials and Methods

### Scaffold Fabrication

The system used to fabricate the scaffolds in this study has been reported previously.^15^ Briefly, poly(ε-caprolactone) (PCL, Mn = 45,000 g/mol; Sigma) was molten at 110 °C for 30 min in a metal heated cartridge. The molten polymer was extruded using 3 bar pressure through a 260 μm diameter nozzle (25G encapsulation needle, DL Technology). A 2 (or 4) mm diameter stainless steel mandrel was attached to the collet grip of a DC motor (Unimat 12 V DC) and powered by a power supply (Voltcraft LPS1153, Conrad). The rotating speed was kept at 1060 rpm. The speed of the printhead over the collector and the number of travels were modified to obtain constructs with a variety of alignments, ranging from printhead speed of 1 to 30 mm/s and number of travels from 2 to 160 times.

### Scaffold Characterization

#### Imaging

Scanning electron microscopy was employed to quantify the fiber thickness and alignment. Samples were cut in half longitudinally, attached onto SEM stubs with the lumen facing up, and gold sputter coated (SC7620, Quorum Technologies). Samples were then imaged in a JSM-IT200 InTouchScope Scanning Electron Microscope (Jeol, Japan). Using ImageJ, the fiber thickness was measured, and the winding angle was assessed by measuring the angle between two adjacent fibers.

#### Mechanical Testing

Mechanical characterization was performed on a mechanical tester (ElectroForce, TA Instruments) with a 45 N load cell (ElectroForce). Samples were mounted on a three-point bend testing system and probed at a rate of 2% strain per second up to a total strain of 160%. The test was captured using a camera (DMC-G3, Panasonic, The Netherlands) with a macrolens (Panagor 90 mm f2.8, Komine, Japan). The hook constant was calculated as the slope of the linear portion from the obtained force–displacement plots.

#### Leak Test

Scaffolds of 2 mm in diameter were immobilized with 1.6 mm barbed plugs and connected via a Luer lock system to a 5, 20, or 50 mL syringe filled with food-dye-colored water (or 5% BSA in PBS solution with food dye). The syringe was connected to a syringe pump (New Era Pump Systems) with a flow rate of 1.2, 10.2, or 25 mL/min. Tests were recorded with the aforementioned camera setup.

#### Suturing

Scaffolds were sutured onto paraformaldehyde-fixed porcine carotid arteries using continuously running 6-0 Prolene sutures (Ethicon, Johnson & Johnson). While being imaged under a stereomicroscope (Olympus), scaffolds were firmly pulled apart from the tissue to illustrate their resistance. Porcine carotid arteries were obtained from the Central Animal Testing Facilities (Maastricht University) through their tissue sharing program from ethically approved studies.

### Cell Seeding of Scaffolds

Bone-marrow-derived human mesenchymal stromal cells (hMSCs) were purchased from Texas A&M Health Science Center, College of Medicine, Institute for Regenerative Medicine (Donor d8011L, female, age 22), and used at passage 5. Cells were expanded in alphaMEM (Gibco) supplemented with 10% FBS (Sigma). HUVECs were purchased from PromoCell as a cryovial of pooled donors. Cells were cultured in EGM2 (PromoCell) and used until passage 5.

Scaffolds were coated with a thin fibrin layer to promote cell adhesion, as described previously.^15^ Briefly, the day before cell seeding, scaffolds were decontaminated by a treatment of 70% ethanol for 30 min and air-dried. Then, 30 μL of fibrinogen was pipetted into the lumen of the tube and allowed to react for 5 min. Samples were then blotted dry onto Whatman paper 1 for 5 min, and subsequently 30 μL of thrombin solution was pipetted into the luminal side. After 5 min reaction time, samples were blot-dried onto Whatman paper 1. To ensure the full polymerization of the thin layer of fibrin, samples were incubated overnight at 37 °C. Scaffolds were then decontaminated again with 70% ethanol for 30 min with mild shaking and then allowed to dry inside a laminar flow hood.

Cells were trypsinized using a standard protocol (trypsin-EDTA 0.25%, Gibco) and counted. Cell concentration was adjusted to 1.05 × 10^6^ cells/mL. Each scaffold was placed into a 0.65 mL Eppendorf tube, and 30 μL of cell suspension was carefully pipetted into the lumen. Tubes were closed and inserted into the “seeding device” (as described elsewhere^15^), placed in an incubator, and rotated 90° every 15 min for 2 h.

To induce a smooth muscle-like phenotype, the medium was switched upon seeding to alphaMEM supplemented with 1% FBS, 5 ng/mL TGF-β1 (PeproTech), and 30 μM ascorbic acid (Sigma).

After 2 weeks of culture, HUVECs were seeded into the luminal side of the scaffold as described above. Scaffolds were placed in tubes, and 30 μL of cell suspension (2 × 10^6^ cells/mL) was carefully pipetted into the lumen and rotated every 15 min for 2 h. Scaffolds were then cultured in the EGM2 medium for 3 days.

### Gene Expression

Three scaffolds were pooled into a 1.5 mL tube with 1 mL of Trizol (Invitrogen) and frozen in liquid nitrogen or immediately processed for RNA extraction. Tubes were thoroughly vortexed until the scaffold was dissolved. Chloroform (200 μL) was added, and tubes were vortexed for 1 min and left to incubate at room temperature for 5 min. Samples were then centrifuged for 15 min at 12,000*g* at 4 °C. The upper phase was collected and transferred into a new tube, where the same volume of isopropanol was added together with 3 μL of Glycoblue (Invitrogen). Samples were stored at −30 °C overnight. The following day, samples were centrifuged for 15 min at 12,000*g* at 4 °C. The supernatant was carefully discarded, and the pellet was washed with 70% ethanol in RNase-free water. Samples were centrifuged once more, ethanol was removed, and pellets were air-dried. Isolated RNA was dissolved in RNase-free water and quantified using a Biodrop (brand). Unless lower amounts of RNA were obtained, 500 ng was retrotranscribed into cDNA using an iScript cDNA synthesis kit (Biorad).

Quantitative real- time PCR was carried out on a BioRad CFX96 instrument (Biorad). cDNA, primers, and iQ SYBR Green Supermix (Biorad) were added into 96-well plates, with a final volume of 10 μL per well. The running protocol consisted of 3 min at 95 °C followed by 40 cycles of 15 s at 95 °C and 30 s at 55 °C and a final melting curve. Results were analyzed using the ΔΔC_t_ method and expressed as 2^–ΔΔCt^. Cells left over after seeding the scaffolds were used as controls. Primers used are described in Table 1 (5′ → 3′).

**Table 1:** Primer Sequences Used for qPCR

GAPDH R	CCATGGTGTCTGAGCGATGT
GAPDH F	CGCTCTCTGCTCCTCCTGTT
COL1a1 F	GAGGGCCAAGACGAAGACATC
COL1a1 R	CAGATCACGTCATCGCACAAC
ACTA2 F	ACGTGGGTGACGAAGCACAG
ACTA2 R	GGGCAACACGAAGCTCATTGTA
CALP F	GCTGTCAGCCGAGGTTAAGAA
CALP R	TGAGGCCGTCCATGAAGTTG
SM22α F	ATGGAGCAGGTGGCTCAGTTC
SM22α R	ACTGCCAAGCTGCCCAAAG

### Immunohistochemistry

Samples were fixed in 4% formaldehyde in PBS for 15 min at room temperature and then stored in PBS at 4 °C until staining was performed. Samples were carefully cut in half perpendicularly using a razor blade (Personna). Samples were blocked and permeabilized with 5% donkey serum in PBS with 0.1% Triton X-100 for 1 h at room temperature. Primary antibody was then added and incubated overnight at 4 °C. Antibodies used and their concentrations were as follows: alpha smooth muscle actin (ab7817, abcam, 1:300), calponin (ab700, abcam, 1:100), and CD31 (ab24590, abcam, 1:300). After three 5 min washes in 0.5% BSA (w/v) in PBS, Alexa488-conjugated secondary antibodies (ThermoFisher) were incubated for 1 h at room temperature while protected from light at a concentration of 1:200 together with Alexa568-conjugated phalloidin (ThermoFisher) at 0.5 μM. After three 5 min washes in 0.5% BSA (w/v) in PBS, samples were incubated with 0.2 μg/mL DAPI in PBS for 15 min and washed once prior to imaging. Imaging was carried out routinely with a Nikon Ti-E microscope; when more detailed images were needed, samples were imaged with a laser scanning confocal microscope (SP8, Leica).

### Biochemical Assays (DNA, GAG, H-Pro)

Cell-seeded scaffolds were digested in 1 mg/mL Proteinase K solution in Tris buffer overnight at 56 °C. DNA quantification was carried out by Hoechst 33258 determination following the manufacturer’s instructions (Sigma). Briefly, a standard curve with known concentrations of calf thymus DNA was performed. Twenty microliters of digested sample or standard was pipetted into a black-bottomed 96-well plate and then mixed with 200 μL of working dye solution, incubated for 5 min in the dark at room temperature, and then analyzed in a plate reader at 360 nm excitation and 460 nm emission (ClarioStar). GAG production was quantified using the 1,9-dimethyl-methylene blue (DMMB) assay. Briefly, a standard curve was performed using known concentrations of chondroitin sulfate (Sigma). Samples and standards were reacted with DMMB 16 mg/L solution, and absorbances at 525 and 595 nm were measured using a plate reader (ClarioStar). Collagen deposition was determined by quantification of hydroxiproline using a chloramine T assay. Briefly, proteinase K digested samples were subjected to a second digestion with concentrated hydrochloric acid at 110 °C. After oxidation with 28.2 mg/mL chloramine T, 0.5 g/mL 4-(dimethylamino)benzaldehyde was added and incubated at 60 °C. Finally, absorbance at 570 nm was measured using a plate reader (ClarioStar). Collagen was estimated from the hydroxiproline content by using a hydroxyproline-to-collagen ratio of 1:7.692.

### Statistical Analysis

All experiments were performed at least in triplicate. GraphPad 9.0 (GraphPad Software, Inc., La Jolla, CA, USA) was used to visualize and analyze the data. When comparing multiple groups, one-way ANOVA and Tukey post hoc analysis were performed. When comparing two groups, a two-tailed Student *t* test was carried out. Differences between groups were deemed significant when *p* < 0.05. All data are presented as the mean ± standard deviation.

## Results

**Figure 1:**
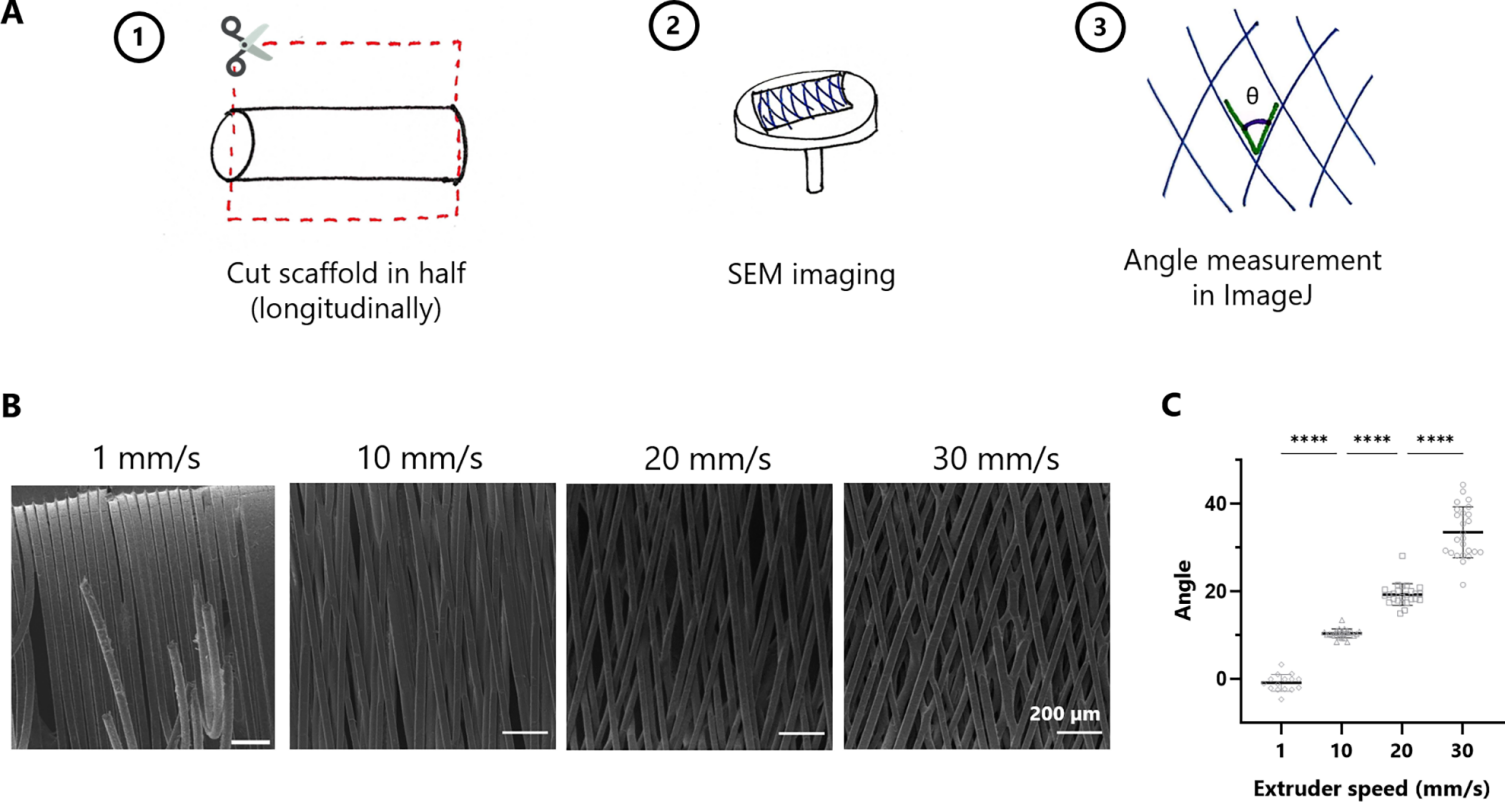
Effect of printhead speed on the scaffold fiber architecture. (A) Schematics showing the procedure used to quantify the angle between fibers. (B) Scanning electron microscopy images of produced scaffolds. (C) Quantification of angle between fibers from SEM images (*n* = 15–25, **** *p* ≤ 0.0001).

Our fabrication method allows fine control over fiber orientation in the circumferential direction (Figure 1). The angle between fibers was quantified from images obtained by SEM. When the printhead was moved at a speed of 1 mm/s, the fibers were deposited parallel to each other (0 angle). At a speed of 10 mm/s, the angle between two fibers was 10.4 ± 1.0°; at a speed of 20 mm/s, the fiber angle was 19.3 ± 2.5°; and at a speed of 30 mm/s, the fiber angle was 33.6 ± 5.8°. The fiber diameter was 31.6 ± 3.9 μm, and it was not affected by the printhead speed. Fiber diameter can also be modified by changing the rotational speed of the collector, as demonstrated in an earlier work.^15^ In this study, this parameter was kept constant at 1060 rpm. For further testing, three fiber alignments were chosen (0, 19, and 33°) and, for easier labeling, were called 1, 20, and 30°.

**Figure 2:**
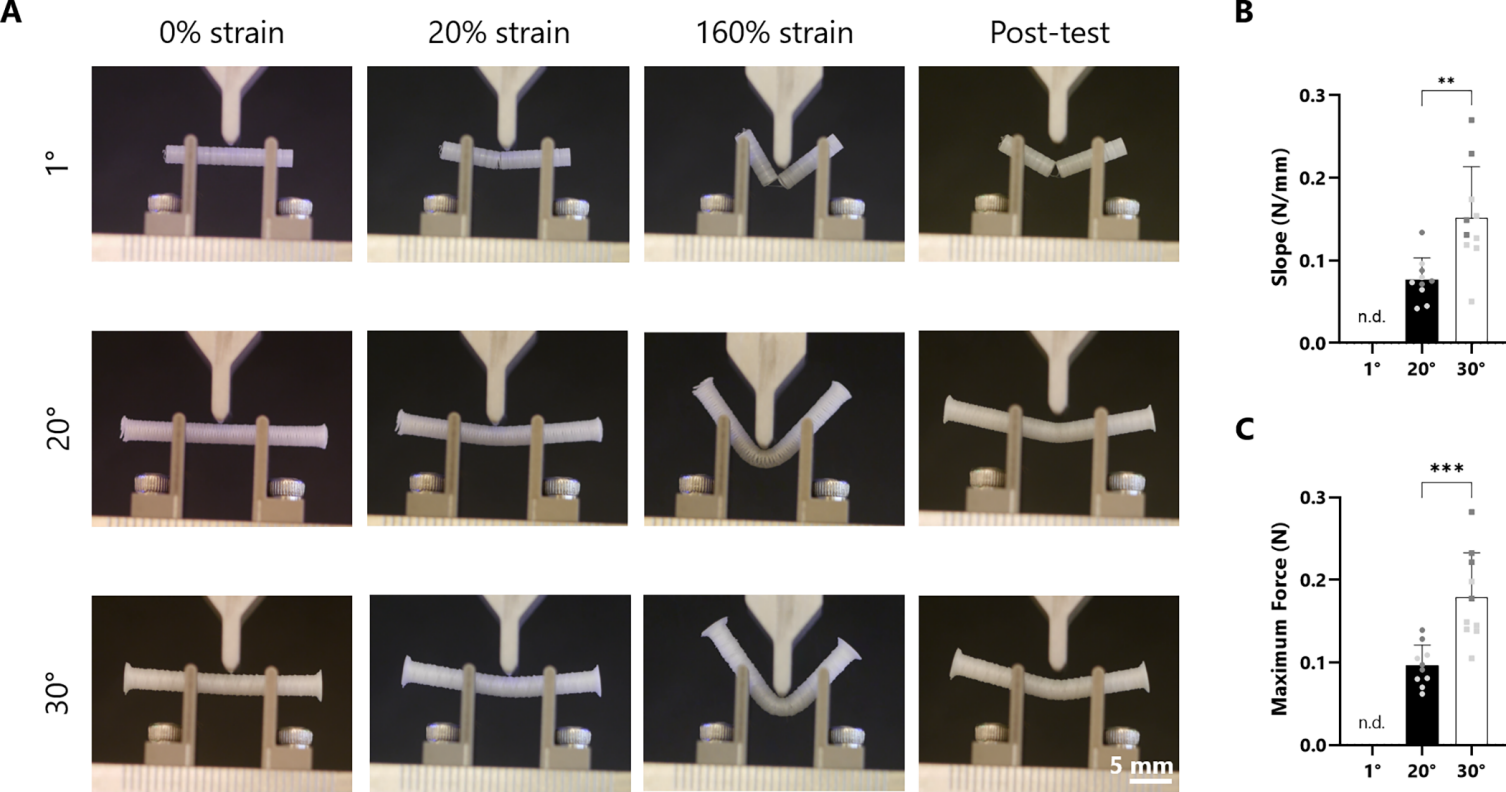
Three-point bending test. (A) Macroscopic imaging at the beginning, at 20% strain, at 160% strain, and post-test. (B) Slope values from force–displacement measurements. (C) Maximum force values from force–displacement measurements. Two shades of gray indicate two separate experiments with a minimum *n* = 4 (total *n* = 10) (** *p* ≤ 0.01, *** *p* ≤ 0.001, n.d. = not determined).

A three-point bending test was performed to assess the mechanical behavior of the produced scaffolds (Figure 2). Video recordings during testing were used to examine defects appearing on the scaffolds. The 1° scaffolds showed a brittle behavior because they already broke at 10% strain. The 20° scaffolds were more robust, displaying no visible defects up to 60% strain, but started presenting clear openings of the fibers after that. The 30° scaffolds had no visible defects at the highest tested strain of 160%. The 20 and 30° scaffolds could be bent up to 160% strain (maximum tested) without kinking or scaffold breakage. At such great strains, 20° scaffolds presented defects and the opening of the fiber mesh, whereas 30° scaffolds remained intact with no noticeable defects. At large strains, scaffolds plastically deformed (as one would expect from PCL) but recovered to a large extent (last column of Figure 2A). From the acquired force–displacement data, the linear region was selected to calculate the slope (equivalent to the hook constant). The maximum load was also quantified. From the quantitative analysis, 30° scaffolds showed significantly higher slope values (0.15 ± 0.06 vs 0.08 ± 0.03 N/mm) (Figure 2B). Furthermore, 30° scaffolds presented a significantly higher maximum force compared to 20° scaffolds (0.18 ± 0.05 N vs 0.10 ± 0.03 N). The 1° samples were extremely delicate and broke easily, and thus data could not be satisfactorily acquired. Samples had noteworthy uniformity in their mechanical behavior (Supplementary Figure 1). Taken together, these results illustrate the impact of fiber alignment on scaffold mechanical properties, with 30° scaffolds being significantly more flexible and resistant, presumably by the fiber entanglement present. The 1° scaffolds had no intertwining of the fibers and had very limited flexibility. Scaffolds of 30° were chosen for further testing as they showed the best mechanical properties.

**Figure 3:**
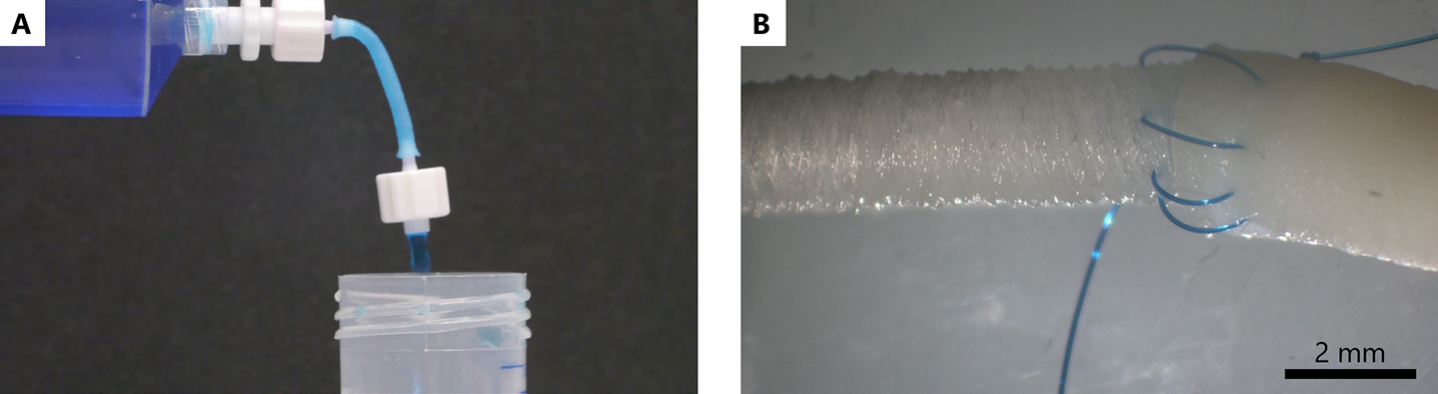
(A) Close-up of the leak test running at 25 mL/min. (B) Scaffold sutured onto the porcine carotid using a continuous running suture (6-0 Prolene).

To test the ability of our scaffolds to hold liquids, Luer lock barbed plugs were introduced at each end of the scaffolds and connected to a syringe mounted on a syringe pump (Figure 3). Water with blue food coloring or a 5% BSA solution in PBS with red coloring was flowed through the scaffolds. The solution with BSA was used as a “proxy” of blood. No leakage was observed at any of the flow rates tested (1.2, 10.2, and 25 mL/min) (Supplementary Videos 1 and 2). Despite the scaffolds being able to hold liquid, they presented high porosity (Supplementary Figure 3). Noteworthily, the scaffolds tested in the leak experiments had been previously used for the three-point bending test, indicating that the high strains applied had not negatively affected the scaffolds.

The 30° scaffolds were sutured onto fixed porcine carotid arteries using 6-0 Prolene sutures (Figure 3B). When firmly pulling the scaffold and the tissue apart, some small gaps could be observed in the scaffold, but these gaps mostly closed after the pulling ceased (Supplementary Videos 3 and 4).

**Figure 4:**
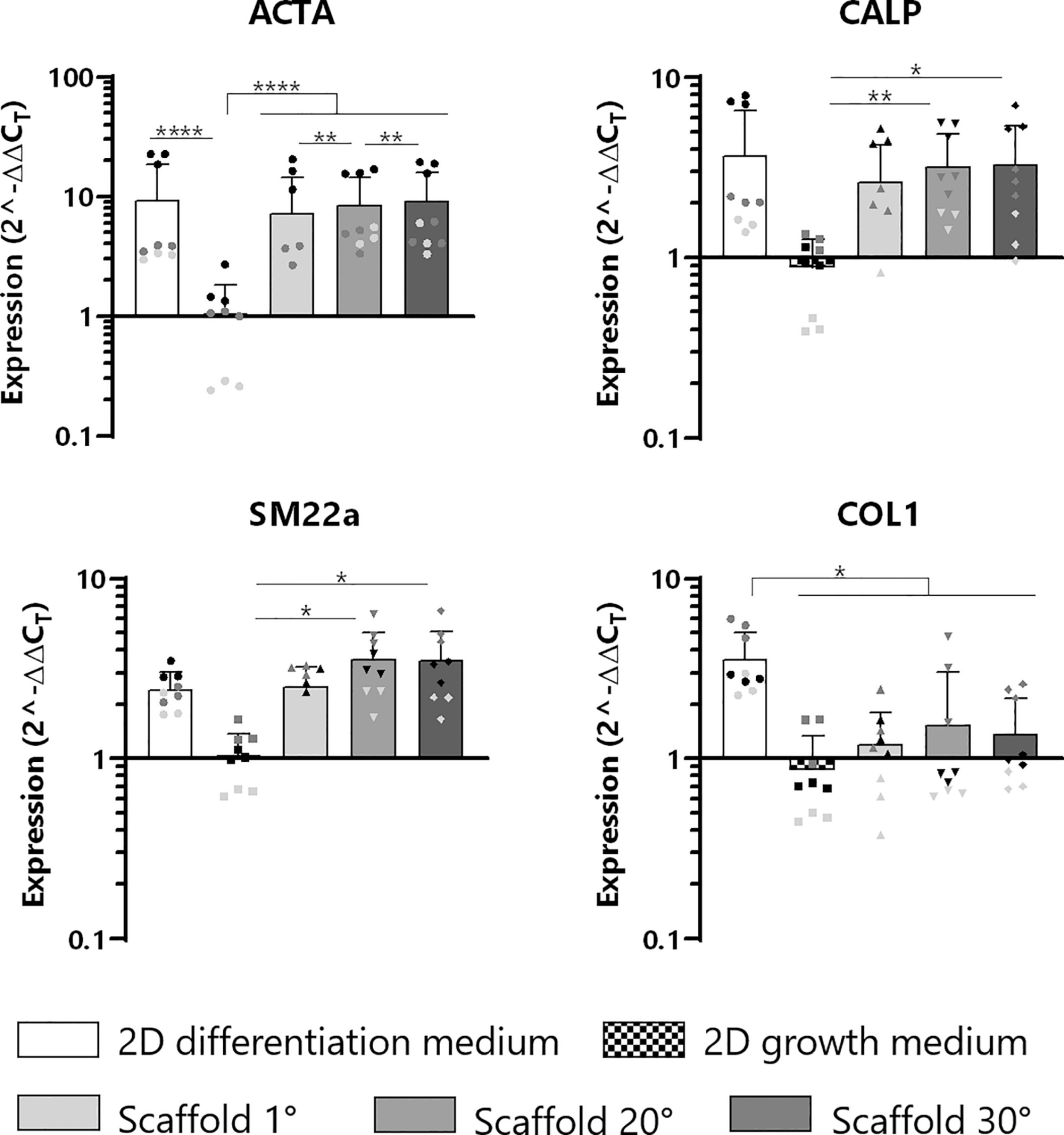
Gene expression of hMSCs grown on scaffolds determined via qPCR (symbols with the same shade of gray indicate the same experiment, *N* = 3, *n* = 9, *p ≤ 0.05, ** *p* ≤ 0.01, *** *p* ≤ 0.001, **** *p* ≤ 0.0001).

Human mesenchymal stromal cells isolated from the bone marrow of healthy donors were seeded in the luminal areas of the scaffolds. The medium used contained low FBS content, TGF-β1, and ascorbic acid to induce a smooth muscle-like phenotype (Supplementary Figure 4). The expression of smooth muscle cell markers was analyzed via quantitative PCR (Figure 4). ACTA2 (the gene for alpha smooth muscle actin) was significantly upregulated when using the differentiation medium in 2D and in the scaffolds. The higher angle between fibers had a positive effect, as 20 and 30° showed significantly higher expression than 1°. CALP and SM22a were significantly upregulated at 20 and 30° compared to the 2D undifferentiated control. However, 2D differentiated and 1° showed a clear upregulation, although it was not statistically significant. Collagen 1 expression was significantly upregulated in the differentiated medium in 2D, but not in the scaffolds’ 3D environment. Taking the gene expression data together, we can conclude that the hMSCs in the scaffolds adopted a smooth-muscle-like phenotype, hinting to a contractile rather than synthetic phenotype.

**Figure 5:**
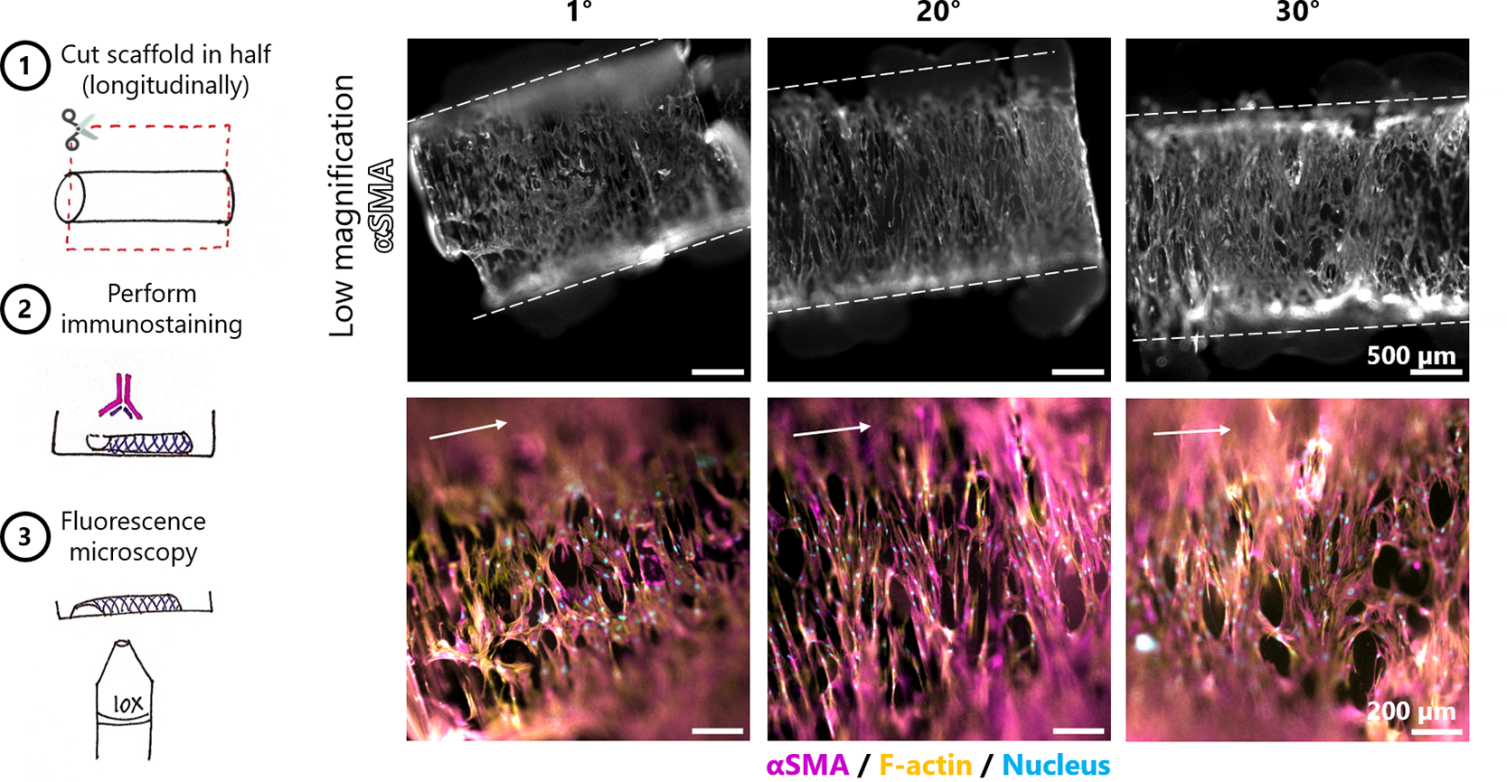
Immunostaining for αSMA of seeded scaffolds after 14 days of culture. Schematics of the staining and imaging procedure (left). The dotted lines indicate the edges of the scaffold, and the arrow indicates the longitudinal axis of the scaffold.

**Figure 6:**
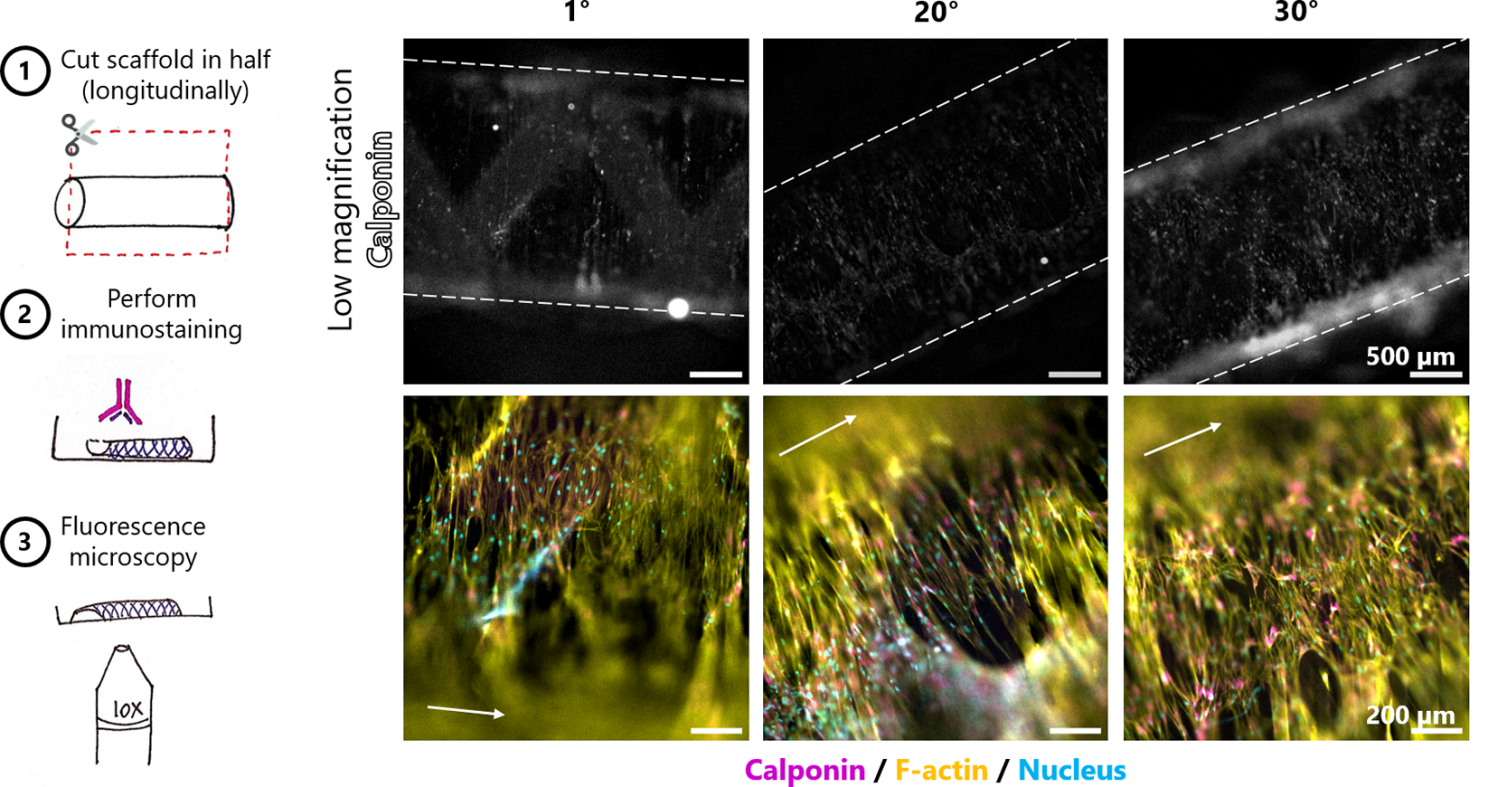
Immunostaining for calponin of seeded scaffolds after 14 days of culture. Schematics of the staining and imaging procedure (left). The dotted lines indicate the edges of the scaffold, and the arrows indicate the longitudinal axis of the scaffold.

Immunostaining confirmed the observations from PCR. Seeded cells showed positive staining for αSMA in all three scaffold architectures at 7 (Supplementary Figure 5) and 14 days (Figure 5). Similarly, calponin staining demonstrated that a majority of the cells expressed this marker at day 14 (Figure 6). The expression of calponin was more prominent in 20 and 30° than 1° scaffolds, with the staining localized to the perinuclear area rather than at the edges and throughout the whole cytoplasm.

**Figure 7:**
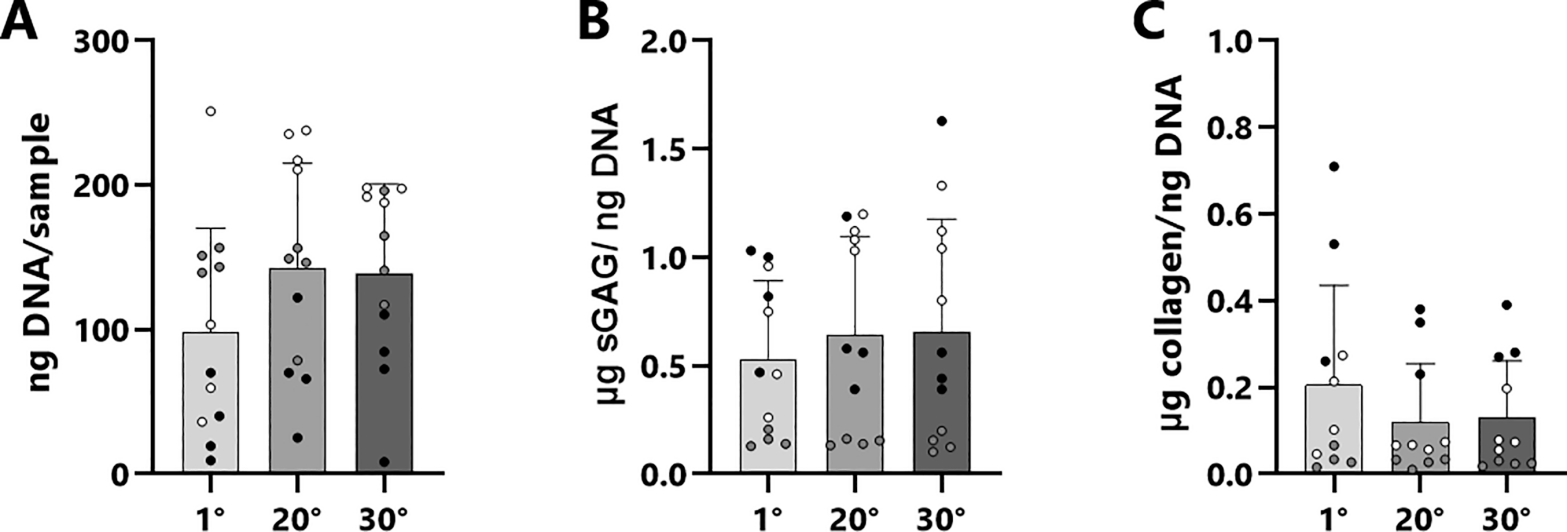
Biochemical quantification of DNA (A), sGAG (B), and collagen (C) after a 14 day culture period.

There did not seem to be much proliferation over the 14 day period (Figure 7A). This could be expected because the used medium contained a very low serum concentration. Deposition of sGAG and collagen was moderate (Figure 7 B,C), which was also seen at the gene level (Figure 4). These quantifications were performed at a rather short culture period of 14 days, which may result in a further increase of ECM production at longer maturation times.

**Figure 8:**
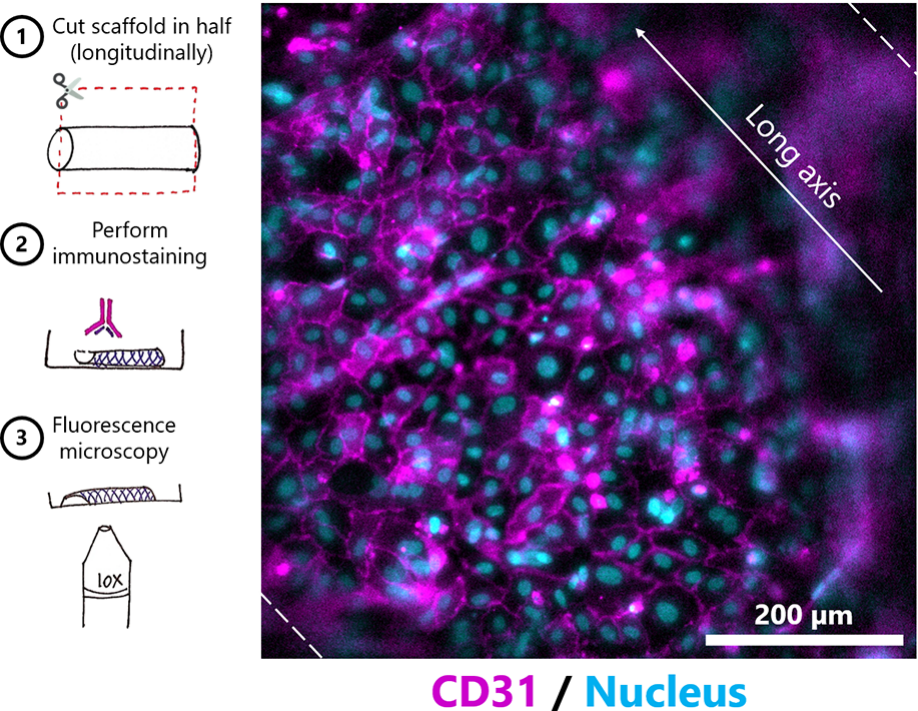
Immunostaining for CD31 of seeded scaffolds in coculture. Schematics of the staining and imaging procedure (left). The dotted lines indicate the edges of the scaffold, and the arrow indicates the longitudinal axis of the scaffold.

HUVECs seeded onto the lumen of repopulated scaffolds attached well and adopted a cobblestone morphology, forming a tightly packed monolayer (Figure 8).

## Discussion

In this study, we described a fabrication method to obtain small diameter tubular scaffolds with potential use as vascular grafts. The scaffolds are composed of circumferentially aligned PCL microfibers, can bend without kinking, are leak free and suturable, and support the growth and differentiation of MSC to vSMC-like cells in the media layer and HUVECs on the intima layer.

Melt spinning, the technique used in this and our previous study,^15^ is simple, requires low “technological” equipment, is versatile in the diameter of the tubes produced, and possesses the ability to control fiber alignment in the circumferential axis. Despite its many benefits, it is not a widespread technique for the fabrication of tubular scaffolds.^15,16^ In a recent publication, Zhi and colleagues described the fabrication of scaffolds in a similar fashion to the one described here.^17^ Their approach was to implant the tubular scaffolds subcutaneously before using them as vascular conduits, in a similar fashion to Geelhoed and colleagues using PEOT/PBT.^9^ The researchers obtained excellent results in lapine, ovine, and canine models, showing no signs of calcification, intimal hyperplasia, or aneurysms.^17^ The scaffolds developed in this current study aim at one-step off-the-shelf use, avoiding an extra intervention and waiting time. Other techniques such as wet spinning have been shown to allow the formation of circumferentially aligned micron-sized fiber scaffolds for vascular applications.^18,19^ This technique, however, requires the use of solvents and an oil/hexane coagulation bath. Melt spinning, the technique used in this study, differs from melt electrowriting (MEW) in that a voltage is applied between the needle and the collector. Most studies using MEW for tubular applications produce scaffolds with extremely large pores (∼150 × 80 μm), and thus leaky, and so need to be combined with electrospinning.^20,21^ Although two techniques needed to be used, the work by Jüngst and colleagues demonstrated excellent scaffold properties. The electrospun lumen could fuse with the deposited MEW fibers and allowed for cell permeability between both layers. Early work by Brown et al. described the fabrication of tubular scaffolds with control over the winding angle using MEW that resemble the ones in this study, with fibers ranging from 20 to 60 μm, using 50 kDa PCL but a larger-diameter (6 mm) collecting mandrel.^22^ Similar work by Pien and colleagues described the fabrication of tubular scaffolds produced by MEW using a photo-cross-linkable acrylate PCL with tunable mechanical properties upon blending with PCL.^23^

Previous research has pointed out the importance of fiber alignment and fiber architecture in the mechanical behavior of tubular scaffolds.^24,25^ Using a three-point bending test, as done in this study, they obtained a variety of scaffolds that went from kinking to bending. In the current study, we describe tubes that bend and do not kink. To achieve a similar behavior, other researchers have had to combine electrospinning with other techniques, such as fused deposition modeling^26^ or MEW.^20,21^ Avoiding kinking is essential to ensure correct blood flow and avoid collapse and wall fusion upon implantation.

In this study, bone-marrow-derived MSCs were employed to populate the scaffolds *in vitro* as they have been demonstrated to be plastic cells, which can differentiate into vascular smooth muscle-like cells.^27,28^ The reduction of serum in the medium and the addition of ascorbic acid and TGF-β1 induced the upregulation of contractile markers αSMA, calponin, and SM22a while reducing the expression of synthetic markers such as collagen 1. Similar results were obtained in PCL tubular scaffolds fabricated via electrospinning and MEW, also in a coculture with endothelial cells.^20,21^

The use of biodegradable polymers has been investigated thoroughly because nondegradable materials such as ePTFE are not suitable for small-diameter grafts due to intima hyperplasia, low endothelialization, and no cell infiltration on the adventitia side and present a high degree of endoluminal thrombus formation.^29,30^ PCL is a widespread material for vascular graft fabrication for its processing ease in electrospinning and melt extrusion-based additive manufacturing techniques.^31^ Early work showed the tendency of PCL to calcify, only visible in long-term studies (≥18 months), which are not as common as mid- and short-term implantation studies (1–6 months). Small calcifications appeared initially only in the intimal hyperplasia area, but then extended transmurally. However, no aneurysms or no ruptures were detected, whereas full endothelialization and patency were achieved.^32^ The rate of degradation needs to come in hand with neotissue formation; enough collagen and elastin need to be deposited to achieve enough mechanical characteristics while the polymer disappears. PCL has long degradation times *in vivo* (>18 months),^33,34^ but degradation is influenced by polymer molecular weight, fiber size, porosity, or crystallinity.^35^

Scaffold porosity can influence cellular infiltration and thus the secretion of enzymes that can increase polymer degradation. The scaffolds developed in this study presented pores sufficiently large for cell infiltration, which are sometimes a problem associated with electrospun mats. In fact, micrometer-sized fibers such as the ones from our scaffolds could be more suitable than nanosized fibers. *In vitro* and *in vivo* research has shown the effect of thicker fibers (micron size) in the polarization toward an M2 (anti-inflammatory) phenotype compared to fibers in the nano range.^36^ The authors hypothesized that electrospinning thicker fibers could lead to bigger pore sizes, and thus increased cell infiltration. Similar results were obtained *in vitro* in PCL-bis-urea (PCL-BU) electrospun mats.^37^

Biodegradable polymers with melting temperatures <200 °C can be processed to fabricate tubular scaffolds with the technique described here, such as PLA or PLCL. Supramolecular polymers such as PCL-BU or PCL-diUPy are ideal candidates for this because they have been used in the cardiovascular space with good *in vivo* results.^38,39^ A recent study using PC(e)-BU electrospun tubes showed a high variability in outcomes from *in vivo* experiments despite the uniformity in fabrication.^40^ This polymer degrades in 1–3 months, which might be too fast to ensure enough neotissue formation, and mismatch of mechanical properties occurs, leading to aneurysms and calcification. The thicker fibers obtained with our technique could lead to a slightly slower degradation and overcome these issues.

To improve *in vivo* responses to these scaffolds, biofunctionalization can be implemented. Attaching peptides on the luminal side can enhance rapid endothelialization, essential to avoid thrombi formation. Candidates for this are laminin-derived YIGSR,^41^ fibronectin-derived REDV,^42^ or SDF-1a-derived SKPVSLSYR.^43^ Researchers have developed simple ways to covalently attach these peptides onto PCL in additive manufactured scaffolds using click chemistry.^44−46^ Another interesting way to increase the “bioactivity” of degradable polymer scaffolds is the incorporation of extracellular vesicles (EVs). This approach has been successful *in vivo* for vascular grafts made of PCL^47^ and silk fibroin.^11^

Future work will focus on the study of the hemocompatibility of these scaffolds, after immobilization of antithrombogenic agents and/or other bioactive molecules to improve fast endothelialization, as mentioned previously. Additionally, testing the leakage from the scaffolds will need to be performed at normal blood pressure, which is a limitation of this current study. Furthermore, we will explore the translation of the methodology used to fabricate scaffolds of other biodegradable thermoplastic polymeric materials and study their mechanical properties and biological behavior.

In conclusion, by tuning processing parameters using the melt spinning technique, we have developed scaffolds with great promise as vascular conduits. Faster translational speed of the printhead yielded constructs with higher fiber intertwinement, which led to the scaffolds being flexible, suturable, and leak-free. By mimicking the ECM disposition of the media layer, the scaffolds supported the growth of a bilayered graft *in vitro*.

## Supporting Information Available

Raw data from mechanical testing, leakage test setup, SEM images for porosity analysis, gene expression to optimize medium, and immunostaining at day 7 (PDF)
Video showing that no leakage was observed (MP4)
Video showing that no leakage was observed (MP4)
Video showing suture retention of the fabricated scaffolds when connected to a carotid artery, despite the occurrence of small gaps that mostly closed after pulling ceased (MP4)
Video at higher magnification showing suture retention of the fabricated scaffolds when connected to a carotid artery, despite the occurrence of small gaps that mostly closed after pulling ceased (MP4)
