# Supplementary material for: Flexible, Suturable, and Leak-free Scaffolds for Vascular Tissue Engineering Using Melt Spinning
Source: ACS Biomater Sci Eng. 2023 Jul 25;9(8):5006–14. doi: 10.1021/acsbiomaterials.3c00535 (PMC10428091; doi:10.1021/acsbiomaterials.3c00535)
Supplement: Supplementary file 1 — ab3c00535_si_001.pdf [file ab3c00535_si_001.pdf]

Supplementary Information

Flexible, suturable and leak-free scaffolds for vascular tissue engineering using melt spinning

Julia Fernández-Pérez, Kenny A. van Kampen, Carlos Mota, Matthew Baker, Lorenzo Moroni*

Department of Complex Tissue Regeneration, MERLN Institute for Technology-Inspired Regenerative Medicine, Maastricht University, Universiteitssingel 40, 6229ER Maastricht, the Netherlands

*Corresponding author.

E-mail address: l.moroni@maastrichtuniversity.nl

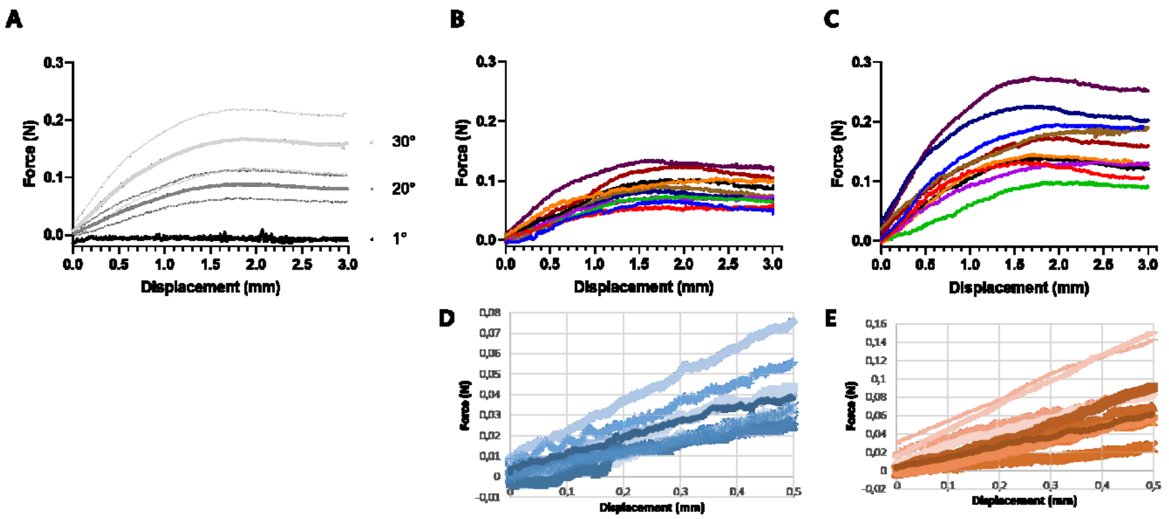

Figure S1. Force-displacement graphs from 3-point bend test results. A) Pooled data. The thick lines indicate the mean and the dotted lines show the standard deviation of 10 samples. For the 1° group, no data could be obtained and only one sample is shown as proof of the kind of un-usable data collected for this group. B) Force-displacement curves of each individual sample of 20° scaffolds (n=10). C) Force-displacement curves of each individual sample of 30° scaffolds (n=10). D) Linear section from which slope is calculated of the Force-displacement curves of each individual sample of 20° scaffolds (n=10). E) Linear section from which slope is calculated of the Force-displacement curves of each individual sample of 30° scaffolds (n=10).

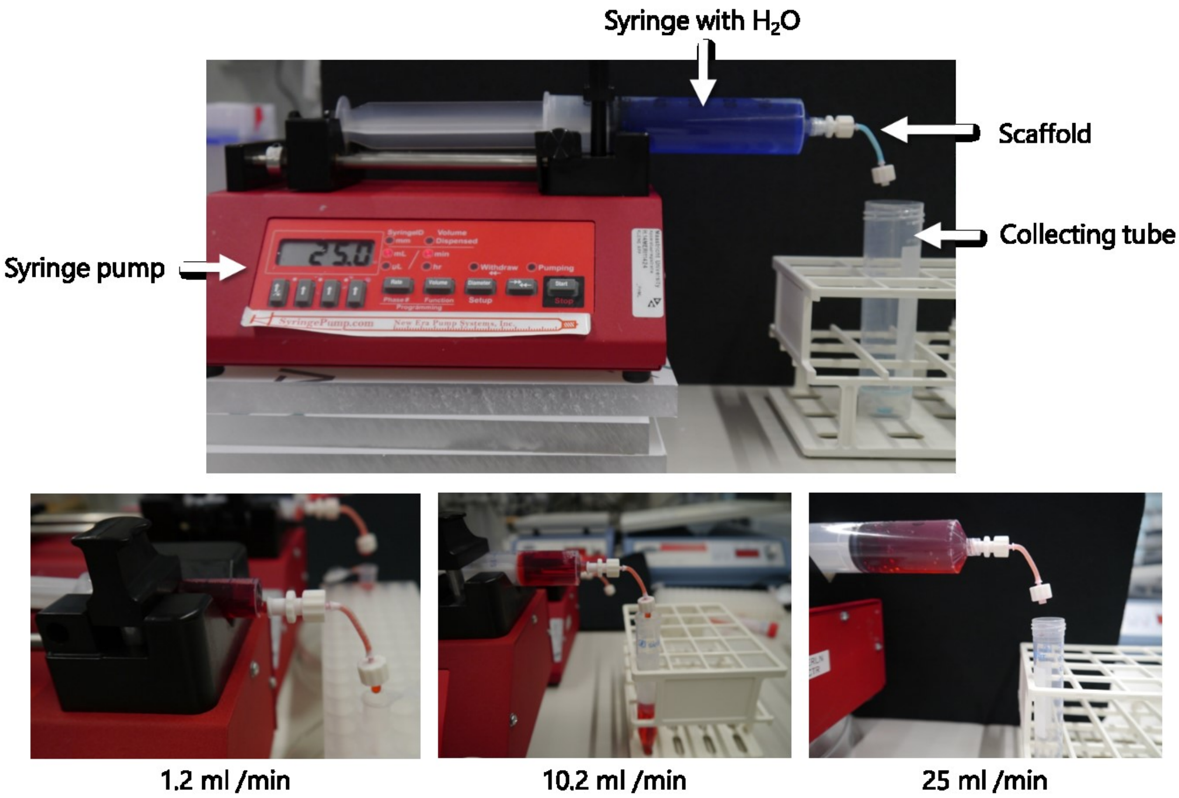

Figure S2. Set-up of leakage test. A variety of flow rates was tested.

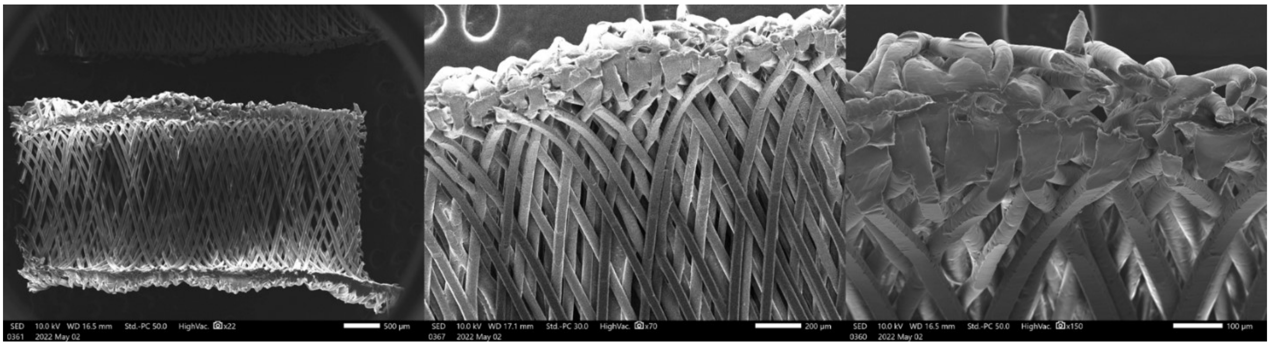

Figure S3. SEM imaging of 30° scaffolds, which illustrate their porosity despite the high amount of fibers. The short and long axes of the dark rhomboid spaces, where the underlying fibers cannot be seen, were measured, and further transformed into a volume taking into account an average fiber size of 31,6 µm. This gave a very rough estimate of pore size, averaging 90.049 μm^3 (range: 42.4 to 145.2 μm^3).

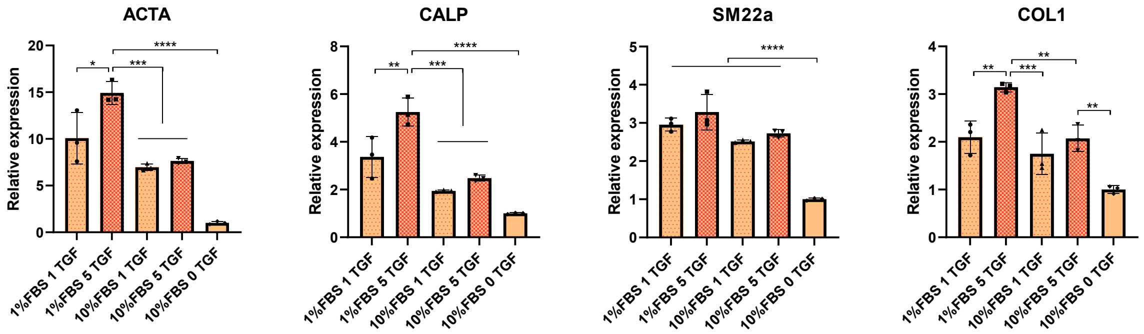

Figure S4. Gene expression of hMSCs determined via qPCR to optimize medium conditions towards an VSMC-like phenotype (*p≤0.05, ** p≤ 0.01, *** p≤0.001, **** p≤0.0001).

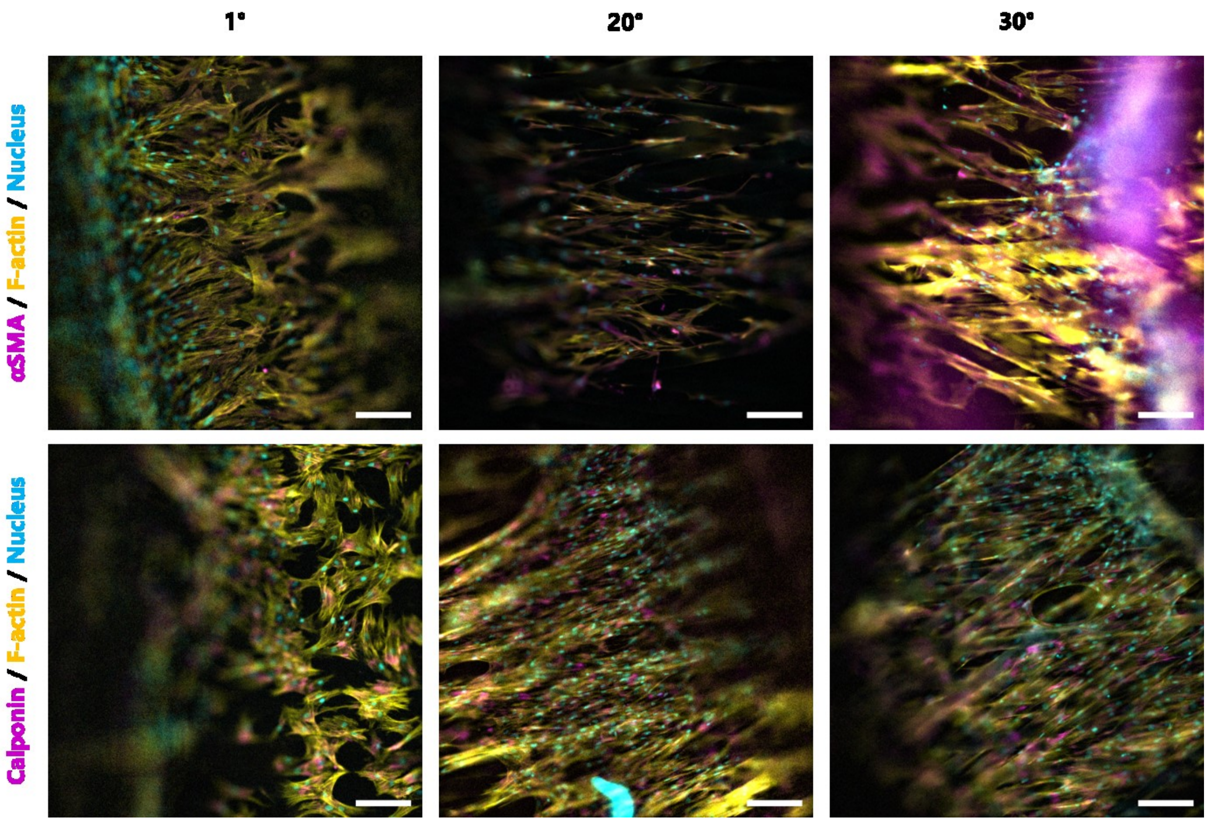

Figure S5. Immunostaining of cells grown on scaffolds after 7 days in culture (scale bar = 200 µm).
